# Der Weg zu Routinedaten aus 16 Notaufnahmen für die sektorenübergreifende Versorgungsforschung: Erfahrungen, Herausforderungen und Lösungsansätze aus der Extraktion pseudonymer Daten für das Projekt INDEED

**DOI:** 10.1007/s00063-021-00879-0

**Published:** 2021-10-28

**Authors:** Antje Fischer-Rosinský, Anna Slagman, Ryan King, Grit Zimmermann, Johannes Drepper, Dominik Brammen, Christian Lüpkes, Thomas Reinhold, Stephanie Roll, Thomas Keil, Martin Möckel, Felix Greiner, INDEED-Projekt, Wilhelm Behringer, Michael Bernhard, Sabine Blaschke, Hans-Jörg Busch, Bernadett Erdmann, Bernhard Flasch, André Gries, Heike Höger-Schmidt, Timo Schöpke, Constanze Schwarz, Rajan Somasundaram, Erik Weidmann, Sebastian Wolfrum, Christian Wrede

**Affiliations:** 1 Notfall- und Akutmedizin (Campus Mitte und Virchow-Klinikum), Charité – Universitätsmedizin Berlin, Augustenburger Platz 1, 13353 Berlin, Deutschland; 2 Institut für Sozialmedizin, Epidemiologie und Gesundheitsökonomie, Charité – Universitätsmedizin Berlin, Berlin, Deutschland; 3 TMF – Technologie- und Methodenplattform für vernetzte medizinische Forschung e. V., Berlin, Deutschland; 4 Universitätsklinik für Anästhesiologie und Intensivtherapie, Otto-von-Guericke-Universität Magdeburg, Magdeburg, Deutschland; 5 OFFIS – Institut für Informatik, Oldenburg, Deutschland; 6 Institut für klinische Epidemiologie und Biometrie, Universität von Würzburg, Würzburg, Deutschland; 7 Landesinstitut für Gesundheit, Bayerisches Landesamt für Gesundheit und Lebensmittelsicherheit, Bad Kissingen, Deutschland; 8 Universitätsklinik für Unfallchirurgie, Otto-von-Guericke-Universität Magdeburg, Magdeburg, Deutschland

**Keywords:** Notfallmedizin, Datenintegration, Sekundärdaten, Dokumentation, Standardisierung, Emergency care, Data integration, Secondary data, Documentation, Standardization

## Abstract

**Hintergrund:**

In Deutschland gibt es bisher keine Gesundheitsberichterstattung zu sektorenübergreifenden Versorgungsverläufen im Kontext einer Notaufnahmeversorgung. Das Projekt INDEED (Inanspruchnahme und sektorenübergreifende Versorgungsmuster von Patienten in Notfallversorgungsstrukturen in Deutschland) erhebt Routinedaten aus 16 Notaufnahmen, die mit ambulanten Abrechnungsdaten der Jahre 2014 bis 2017 personenbezogen zusammengeführt werden.

**Ziel der Arbeit:**

Die methodischen Herausforderungen der Planung der internen Zusammenführung von klinischen und administrativen Routinedaten aus Notaufnahmen in Deutschland bis zur finalen Datenextraktion werden hier gemeinsam mit Lösungsansätzen dargestellt.

**Methodik:**

Die Auswahl der Notaufnahmedaten erfolgte in einem iterativen Prozess unter Berücksichtigung der Forschungsfragen, medizinischen Relevanz und angenommenen Datenverfügbarkeit. Nach einer Vorbereitungsphase zur Klärung der Rahmenbedingungen (u. a. Datenschutz, Ethik), zur Prüfung von Testdaten und ggf. Korrekturen, erfolgte die verschlüsselte und pseudonyme Datenausleitung.

**Ergebnisse:**

Die Daten der 16 kooperierenden Notaufnahmen stammten in der Regel aus dem Notaufnahme- und dem Krankenhausinformationssystem. Die Datenlage war sehr heterogen. Nicht alle Variablen waren in jeder Notaufnahme verfügbar, da sie beispielsweise nicht standardisiert und digital vorlagen oder der Extraktionsaufwand als zu hoch bewertet wurde.

**Schlussfolgerung:**

Relevante Daten aus Notaufnahmen liegen unterschiedlich strukturiert und in mehreren IT-Systemen vor. Die notwendige Bildung eines klinikübergreifenden vergleichbaren Datensatzes erfordert erhebliche Ressourcen auf Seiten der Kliniken sowie der datenaufbereitenden Stelle. Dies muss für zukünftige Projekte großzügig kalkuliert werden.

## Abstract (en)

**Background:**

In Germany there is currently no health reporting on cross-sectoral care patterns in the context of an emergency department care treatment. The INDEED project (Utilization and trans-sectoral patterns of care for patients admitted to emergency departments in Germany) collects routine data from 16 emergency departments, which are later merged with outpatient billing data from 2014 to 2017 on an individual level.

**Aim:**

The methodological challenges in planning of the internal merging of routine clinical and administrative data from emergency departments in Germany up to the final data extraction are presented together with possible solution approaches.

**Methods:**

Data were selected in an iterative process according to the research questions, medical relevance, and assumed data availability. After a preparatory phase to clarify formalities (including data protection, ethics), review test data and correct if necessary, the encrypted and pseudonymous data extraction was performed.

**Results:**

Data from the 16 cooperating emergency departments came mostly from the emergency department and hospital information systems. There was considerable heterogeneity in the data. Not all variables were available in every emergency department because, for example, they were not standardized and digitally available or the extraction effort was judged to be too high.

**Conclusion:**

Relevant data from emergency departments are stored in different structures and in several IT systems. Thus, the creation of a harmonized data set requires considerable resources on the part of the hospital as well as the data processing unit. This needs to be generously calculated for future projects.

## Hintergrund und Fragestellung

Die klinische Notfallversorgung in Deutschland ist mit derzeit rund 21 Mio. [22] Behandlungen pro Jahr einer steigenden Inanspruchnahme ausgesetzt, was eine angemessene Versorgung akuter Notfälle zunehmend erschwert [20, 21]. Mögliche Folgen sind eine Patientenwohlgefährdung durch die steigende Belastung des Personals und die relative Verknappung materieller und räumlicher Ressourcen [2, 23].

Für Notaufnahmen in Deutschland gibt es mit dem Datensatz „Notaufnahme“ der DIVI zwar einen Standard für die klinische Dokumentation [15], der bislang aber nur Empfehlungscharakter hat. Durch die Position der Notaufnahmen an der Schnittstelle zwischen ambulanter und stationärer Versorgung sowie die nichteindeutige Identifikation von Notaufnahmen in Abrechnungsdaten [12] fehlen bisher Daten zum sektorenübergreifenden Versorgungsgeschehen im Kontext der Notfallversorgung in Deutschland. Das vom Innovationsfonds geförderte Projekt INDEED (Kennzeichen 01VSF16044) hat das Ziel, die „Inanspruchnahme und sektorenübergreifenden Versorgungsmuster von Patienten in Notfallversorgungsstrukturen in Deutschland“ zu charakterisieren. Dazu wird eine pseudonyme, personenbezogene Verknüpfung von Routinedaten aus Notaufnahmen mit Daten aus der kassenärztlichen Versorgung (KV) in den zwei Jahren vor bis ein Jahr nach dem Index-Notaufnahmekontakt aus dem Kalenderjahr 2016 erfolgen.

In diesem Beitrag wird das Vorgehen, der Zeitverlauf sowie die Herausforderungen in Bezug auf die Routinedatenextraktion in den beteiligten Notaufnahmestandorten dargestellt. Dabei handelt es sich neben klinischen und administrativen Daten auch um Angaben aus einem sich ggf. anschließenden stationären Aufenthalt.

## Methodik

### Rekrutierung der Kooperationskliniken und Einschlusskriterien

Bereits in der Antragsphase des Projektes wurde mit der Rekrutierung von Notaufnahmen begonnen. Die Ansprache potenzieller Kooperationskliniken erfolgte in bestehenden Netzwerken, wie z. B. dem AKTIN-Projekt [4], sowie auf Veranstaltungen der einschlägigen medizinischen Fachgesellschaften. Unbedingte Voraussetzung für die Teilnahme war eine elektronische Datenerhebung in der Notaufnahme im Jahr 2016. Zusätzlich wurden die datenschutzrechtlichen Voraussetzungen in den jeweiligen Bundesländern eruiert. Wichtig war hier ein vorliegender und ausreichender gesetzlicher Erlaubnistatbestand für die Verarbeitung der retrospektiven klinischen Daten ohne die Möglichkeit der Einholung von Einwilligungen der betroffenen Patienten. Ziel war eine möglichst deutschlandweite Rekrutierung von 15 bis 20 Notaufnahmen unterschiedlicher Größe.

In INDEED werden nur Daten von Patienten erfasst, welche im Kalenderjahr 2014 volljährig waren. Ausgeschlossen wurden Patienten mit *eindeutig* privatem Versicherungsstatus sowie Fälle im Verantwortungsbereich der gesetzlichen Unfallversicherung.

### Ethik- und Datenschutzaspekte für die Ausleitung von Personenpseudonymen

Ein positives Ethikvotum der Charité liegt seit Juni 2017 vor (Antragsnummer EA4/086/17). Die Studie ist im Deutschen Register für Klinische Studien registriert (DRKS00022969).

**Figure 1:**
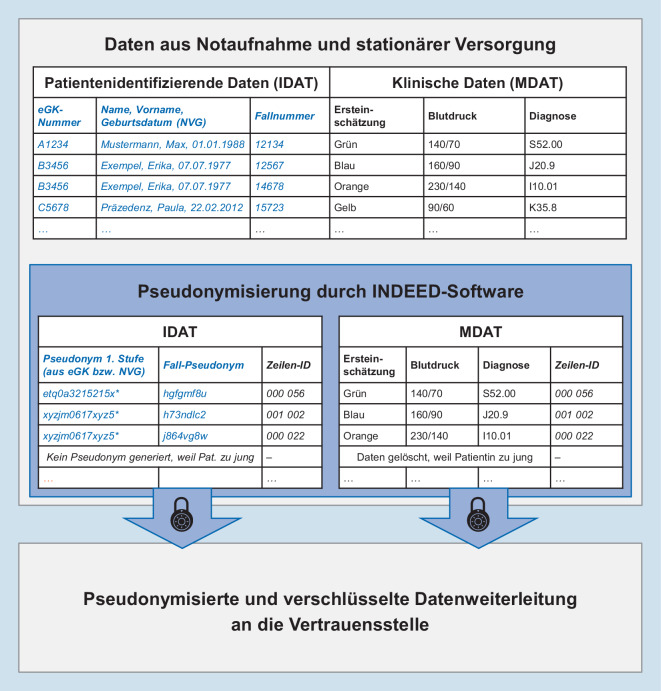

Die Verknüpfung der Notaufnahmedaten mit den Daten aus der kassenärztlichen Versorgung erfolgt über personenbezogene Pseudonyme in einer Vertrauensstelle. Das primäre Pseudonym wird aus der Versichertennummer der gesetzlichen Krankenkasse (eGK-Nummer) gebildet. Zusätzlich wird ein sekundäres Pseudonym auf Basis von Name, Vorname und Geburtsdatum (NVG) erstellt. Zur Generierung der Pseudonyme, Verschlüsselung und Übermittlung der finalen Datensätze an die Vertrauensstelle wurde eine Software speziell für die Bedarfe des Projektes entwickelt [8]. Diese Software, die direkt in den Kliniken zum Einsatz kam, prüft zusätzlich, ob das Alter den Einschlusskriterien entspricht, und löscht ggf. die nicht eligiblen Fälle (Abb. 1). Dafür wurde ein umfangreiches Datenschutzkonzept erstellt, welches eine positive Stellungnahme der AG Datenschutz der TMF – Technologie- und Methodenplattform für die vernetzte medizinische Forschung e. V. (14.02.2018) sowie die Zustimmung der behördlichen Datenschutzbeauftragten der Charité – Universitätsmedizin Berlin (April 2018, Zeichen 565/17/ST3) erhielt. Diese Dokumente bildeten die Basis für die benötigten Datenschutzvoten der einzelnen Kliniken sowie zum Teil erforderlicher behördlicher Genehmigungen aus zuständigen Landesministerien.

### Auswahl der zu extrahierenden Daten

Die Auswahl der Variablen erfolgte in einem interdisziplinären Expertengremium aus Klinikern, Versorgungsforschern, Statistikern, Epidemiologen, Datenschutzexperten und IT-Mitarbeitern in einem iterativen Prozess unter Berücksichtigung folgender Aspekte: Bezug zu den Forschungsfragen, medizinische Relevanz, vorhandene Datensatzdefinitionen (z. B. Datensatz „Notaufnahme“ der DIVI [15], Vorstellungsgründe nach CEDIS [11]), vermutete elektronische Verfügbarkeit sowie Datensparsamkeit. Zur Förderung der Kooperationsbereitschaft der Notaufnahmen wurde auf die Definition eines Minimaldatensatzes verzichtet.

### Vorbereitungen zur Datenextraktion

Parallel zu den administrativen Prozessen wurden die Dokumentationsstandards vor Ort mit den Anforderungen des Projektes abgeglichen sowie eine Extraktion und Prüfung von Testdatensätzen vorgenommen.

Für die individuelle Datenverknüpfung mit den KV-Daten muss sowohl ein Personenbezug als auch für den Fall von mehreren Notaufnahmekontakten im Jahr 2016 ein Fallbezug sichergestellt sein. Zur Aufwandsminimierung vor Ort konnte die Datenlieferung in mehreren Einzeldateien (z. B. Notaufnahmedaten, Laborbefunde, Bildgebung und stationärer Aufenthalt) erfolgen. Weiterhin musste sichergestellt sein, dass diese Dateien im zentralen Datenmanagement des Projektes über die pseudonymisierte klinikinterne Fallnummer korrekt zugeordnet werden können. Mindestens in einer „Stammdatei“ wurde daher immer der Bezug zwischen Fallpseudonym und Personenpseudonym hergestellt.

Die Testdatensätze sowie die endgültigen Gesamtdatensätze wurden im Dialog mit den zuständigen Mitarbeitern direkt in den Notaufnahmen bzw. per Videokonferenz systematisch geprüft. Zum Termin der Datenausleitung waren grundsätzlich zwei Projektmitarbeiter von INDEED vor Ort, um die Datenbestände nach dem Vier-Augen-Prinzip einer finalen Qualitätskontrolle zu unterziehen. Dazu wurde Microsoft® Excel® (Microsoft Corporation, Redmond, WA, USA) genutzt. Eine erste, grobe Plausibilitätskontrolle erfolgte über die Betrachtung spezifischer Häufigkeiten beispielsweise in Bezug auf eine ca. hälftige Geschlechtsverteilung, stationäre Aufnahmequote zwischen 30 und 45 % [19], Stufen der Ersteinschätzung [18] und monatliche Fallzahlschwankungen als Hinweis auf unvollständige Zeiträume. Die Verknüpfbarkeit mehrerer Tabellen über die Fallnummer wurde stichprobenartig überprüft bzw. die Verknüpfung direkt vor Ort vorgenommen. Bei erkannten Unstimmigkeiten wurden – sofern möglich – notwendige Korrekturen direkt vor der finalen Datenausleitung durchgeführt.

## Ergebnisse

### Vorbereitung der Kooperation mit den Notaufnahmen bis Datenausleitung

**Figure 2:**
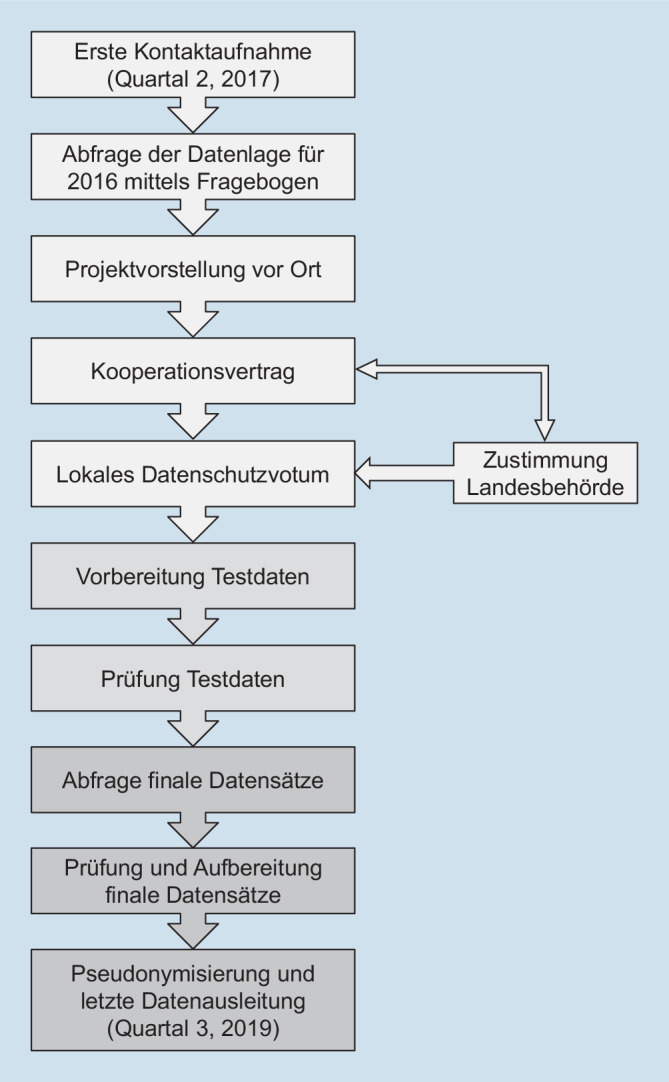

In Abb. 2 ist der Prozess von Kontaktaufnahme mit den potenziell beteiligten Notaufnahmen bis zur finalen Datenausleitung schematisch dargestellt.

Mit 16 Notaufnahmen aus acht Bundesländern (und damit acht KV-Bereichen) wurde das Rekrutierungsziel von 15–20 Notaufnahmen erreicht [9]. Insgesamt wurden mit 31 Kliniken Kooperationsgespräche geführt.

Folgende Gründe führten zum Ausschluss bzw. Nichtteilnahme der Kliniken (Mehrfachnennungen möglich):

- 4 Mal: Elektronische Dokumentation in der Notaufnahme für 2016 nicht ausreichend
- 4 Mal: Personelle Umstrukturierungen im Projektzeitraum
- 3 Mal: Fehlende personelle Ressourcen, insbesondere bei der Klinik-IT
- 2 Mal: Negative Einschätzung des Vorhabens durch den betrieblichen Datenschutzbeauftragten der Klinik
- 2 Mal: Angebotene Aufwandsentschädigung in Höhe von 10.000 € von Klinik als zu gering empfunden
- 1 Mal: Unvereinbarkeit der Herausgabe von pseudonymisierten Krankenhausdaten mit Landesgesetzgebung (Bayern) trotz konstruktiver Bearbeitung durch die zuständige Landesbeauftragte für Datenschutz
- 1 Mal: Grundsätzliche Ablehnung durch die Geschäftsführung

Die teilnehmenden Kliniken wurden – sofern gewünscht – beim Einholen des lokalen Ethikvotums und der Abstimmung mit dem lokalen Datenschutzbeauftragten oder der zuständigen Behörde auf Landesebene unterstützt. Zusätzlichen Aufwand verursachte die parallele Einführung der Datenschutzgrundverordnung (DSGVO) der Europäischen Union im Mai 2018. In Brandenburg und Thüringen musste zudem das jeweils zuständige Landesministerium das Vorhaben bewilligen; in Brandenburg wurde zusätzlich eine Datenschutzfolgeabschätzung gefordert.

### Finale Variablenliste

**Figure 3:**
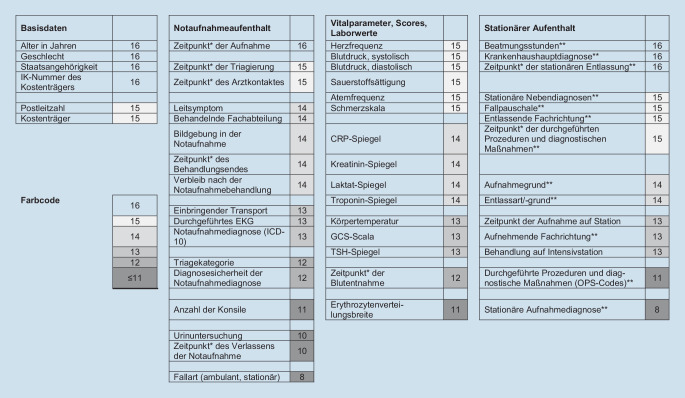

Der konsentierte Datensatz besteht aus 60 Variablen in vier Kategorien: Basisdaten zum Patienten, Daten des Notaufnahmeaufenthaltes (z. B. Ersteinschätzung, Prozessdaten, spezifische Untersuchungen), Vitalparameter, Scores und Laborwerte sowie Daten aus einem sich eventuell anschließenden stationären Aufenthalt (Abb. 3, [9]).

Folgende Änderungen wurden im Vergleich zu der initialen Variablenliste durchgeführt:

- Reduktion der Laborparameter von 36 auf die sechs relevantesten Werte, welche Rückschlüsse auf die Erkrankungsschwere zulassen bzw. Bestandteil klinischer Scores sind.
- Abfrage der Glascow-Coma-Scale nur als Summenscore ohne die drei Einzelkategorien.
- Die Abfrage nach dem Vorliegen eines Einweisungsscheines wurde gestrichen, da die elektronische Verfügbarkeit als unzureichend bewertet wurde.
- Diagnostische Maßnahmen im Notaufnahmekontext wurden aufgrund der unterschiedlichen Dokumentationsroutinen auf ausgewählte bildgebende Verfahren (Röntgen, Sonographie, Computertomographie, Magnetresonanztomographie), Urinuntersuchung und Elektrokardiogramm beschränkt.
- Reduktion auf sieben Zeitstempel (d. h. Datum und Uhrzeit), insbesondere Verzicht der Zeitstempel bei den diagnostischen Prozeduren (s. voriger Punkt).
- Klinische Notaufnahmediagnosen liegen nicht immer vor, da in Notaufnahmen keine direkte Pflicht zur Erhebung von kodierten Diagnosen besteht. Als Surrogat wurden daher für stationäre Fälle die Aufnahmediagnosen nach §301 Sozialgesetzbuch (SGB) V und für ambulante Fälle die Abrechnungsdiagnose(n) nach §295 SGB V ergänzt [12].
- Der Name der Krankenkasse (Freitext) wurde zur Validierung um das Institutskennzeichen ergänzt.
- Ebenfalls ergänzt wurde die pseudonymisierte Betriebsstättennummer (BSNR) der Notaufnahme gemäß kassenärztlicher Abrechnung [14], damit eine Zuordnung des Leistungserbringers über die BSNR in den KV-Daten möglich ist.

### Übermittelte Daten

Die Datenextraktion wurde Anfang September 2019 und damit neun Monate nach dem ursprünglichen Plan (01.12.2018) beendet. Hauptgründe für die Verzögerung waren die langwierigen notwendigen Abstimmungen mit den Kliniken, sehr aufwändige und föderal geregelte Datenschutzanforderungen und Verzögerungen bei der Datenausleitung selbst. Für Letztere wurde pro Klinik ein ganzer Arbeitstag aufgewendet, was bei Einbezug aller involvierten lokalen Mitarbeiter einen erheblichen terminlichen Abstimmungsaufwand erforderte.

Nicht alle Variablen waren in den einzelnen Notaufnahmen für die Datenextraktion verfügbar (Abb. 3). Folgende Gründe wurden dafür ermittelt: Die Variable wurde generell nicht dokumentiert, nicht immer elektronisch dokumentiert, war nicht strukturiert abfragbar (Freitext), oder der Aufwand für die Extraktion wurde als zu hoch angesehen (z. B. „Anzahl der Konsile“ nicht numerisch dokumentiert, sondern hätte durch Sichtung aller Dokumente händisch ermittelt werden müssen).

Die Abfrage der Daten eines sich anschließenden stationären Aufenthaltes war dagegen standardisiert umsetzbar, da außer dem „Zeitpunkt der stationären Verlegung“ und „Aufenthalt auf der Intensivstation“ alle Variablen den gesetzlichen Datensatzdefinitionen gemäß § 301 SGB V bzw. §21 Krankenhausentgeltgesetz entsprechen [5, 6].

**Figure 4:**
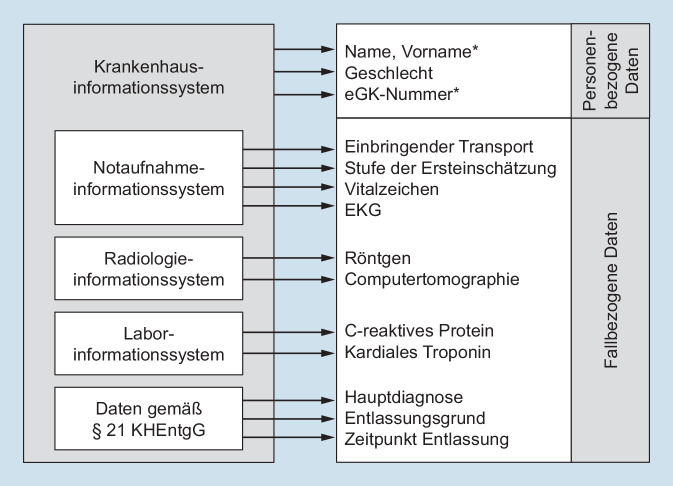

Die Notaufnahmedaten stammten grundsätzlich aus mindestens zwei Systemen, in der Regel dem Notaufnahmeinformationssystem (Emergency Department Information System [EDIS]) und dem Krankenhausinformationssystem (KIS). Je nach Anbieter des IT-Systems, lokaler Konfiguration bzw. Integration mussten weitere Systeme abgefragt werden, insbesondere Labor- und Radiologieinformationssysteme (Abb. 4). Die Fallnummer konnte in bestimmten EDIS durch Hinzufügung von Ziffern länger als im KIS sein, daher musste vor der softwarebasierten Pseudonymisierung eine Korrektur erfolgen. Für die Subsysteme wurden teilweise neben der Fallnummer weitere Strategien zur Auswahl und Verknüpfung der relevanten Daten benötigt, beispielsweise die interne Kostenstelle der Notaufnahme und/oder der Zeitpunkt einer Anforderung.

### Kooperation mit den Notaufnahmen

Die personelle Ausstattung, Strukturen und weitere Ressourcen waren in den kooperierenden Notaufnahmen sehr heterogen. Generell war ein interprofessionelles Vorgehen notwendig, um die Datenausleitung erfolgreich abzuschließen. Involviert wurden dabei klinisches ärztliches und pflegerisches Personal, Wissenschaftler sowie Mitarbeiter der IT-Abteilung und des Controllings. Weiterhin wurde bei fünf Kliniken der EDIS-Hersteller zur Programmierung einer einheitlichen Datenabfrage eingebunden. Hier konnten bestehende Kontakte aus dem AKTIN-Projekt genutzt werden [4].

## Diskussion

Im Projekt INDEED wurden erstmalig für eine multizentrische Studie fallbezogene klinische und administrative Daten aus 16 Notaufnahmen und dem Notaufnahmeaufenthalt sich ggf. anschließendem stationären Aufenthalt extrahiert und zusammengeführt. In Verbindung mit den KV-Daten wurde ein Datenkörper geschaffen, welcher Analysen für die sektorenübergreifende Versorgungsforschung zu Notfallbehandlungen ermöglicht. Neben den aufwändigen Belangen des Datenschutzes wurden dafür folgende Herausforderungen gemeistert:

1. umfangreiche administrative Vorbereitung,
2. interprofessionelle Kommunikation mit Stakeholdern in den Kliniken,
3. Datenaufbereitung in der Klinik,
4. Datenprüfung vor Ort und
5. datenschutzkonforme Datenausleitung.

### Datenschutz

Die heterogenen datenschutzrechtlichen Rahmenbedingungen in den einzelnen Bundesländern führten zu erheblichen Verzögerungen bei der Abstimmung mit Kliniken und Behörden. Die bundeslandspezifische Gesetzeslage verhinderte zudem eine deutschlandweite Rekrutierung von Kliniken [3]. Eine deutschlandweite und trägerübergreifende einheitliche Verfahrensweise wäre wünschenswert, würde aber einen einheitlichen Rechtsrahmen voraussetzen. Durch die Einbindung des Konsortialpartners TMF mit Expertise auf dem Gebiet des Datenschutzes in der medizinischen Forschung und speziell Großprojekten konnten die Verzögerungen eingegrenzt werden. Das Datenschutzkonzept von INDEED ist für Folgeprojekte verfügbar [13] und erfüllt so eines der Projektziele bezüglich der Entwicklung von möglichst generischen Lösungen für den Aufbau von Forschungsinfrastrukturen.

### Entwicklung der Variablenliste

Die Variablenliste ist bis zum Abschluss der Datenausleitung einige Präzisierungen schuldig geblieben, beispielsweise die Operationalisierung der Zahl der behandelnden Fachrichtungen oder einzelner Zeitstempel. Eine konsequente Nutzung von vorhandenen einschlägigen Datensatzbeschreibungen (Datensatz „Notaufnahme“ der DIVI, § 21 KHEntgG) ist zukünftig geboten. Dies verringert den Interpretationsspielraum und sorgt für einen homogeneren Datenbestand. Es hat sich gezeigt, dass Dokumentationsstandards [17] in der Praxis noch nicht flächendeckend bestehen. In anderen Ländern hingegen (wie beispielsweise Australien) ist diese einheitliche medizinische Dokumentation bereits gesetzlich vorgeschrieben [1]. Dadurch kann für unterschiedliche Ziele auf standardisierte Daten zurückgegriffen werden, z. B. Indikatoren zur Messung der Behandlungsqualität, klinikübergreifendes Benchmarking und Versorgungsforschung. Durch die Verpflichtung zur Erhebung eines praxisorientierten und standardisierten Kerndatensatzes zur Notaufnahmeversorgung könnte möglicherweise eine bessere und flächendeckendere Umsetzung in der Praxis erzielt werden [16].

### Ressourcen

Hinsichtlich der Notaufnahmedatenextraktion sind folgende Abwägungen zur Erreichung bestmöglicher Datenqualität zu treffen: Die Übermittlung mehrerer unterschiedlich aufgebauter Datentabellen aus einer Klinik erleichtert zwar die Datenextraktion vor Ort, kann den Datenbankaufbau in der Vertrauensstelle und im zentralen Datenmanagement allerdings erschweren. Bei erhöhten Anforderungen an die Datenaufbereitung in den Klinken sollte jedoch beachtet werden, dass dabei auftretende Fehler später ggf. nicht nachvollziehbar sind und nicht mehr behoben werden können. Besonders zu achten ist auf die Struktur der klinikinternen Fallnummer, da diese die Fallzusammenführung zwischen den verschiedenen Dateien einer Klinik gewährleistet. Zusammengefasst muss das Monitoring der Prozesse den vor Ort vorhandenen Datenmanagement-Kompetenzen angepasst werden.

Der gesamte Prozess zwischen erster Kontaktaufnahme mit den Notaufnahme- und Klinikleitungen, Erfüllung der Formalitäten und Extraktion der Datensätze aus den Kliniken hat sich über ca. 24 Monate hingezogen und überstieg damit deutlich die im Antrag kalkulierte Dauer um neun Monate. Derartige Ergebnisse werden kaum publiziert. Eine Ausnahme ist Eichler et al., die exemplarisch für das Einholen der Ethikvoten in einer multizentrischen Studie von einer starken Verzögerung berichten [7]. Dies ist hinsichtlich der Projektlaufzeit und Personalplanung zu beachten. Die Aufwandsentschädigung von 10.000 € war für einzelne Kliniken nicht kostendeckend. Die Datenabfrage und -aufbereitung musste durch interne Mitarbeiter zusätzlich zu deren eigentlicher Tätigkeit geleistet werden. Dies reduziert die Kooperationsmöglichkeiten mit Kliniken ohne etablierte Forschungsstrukturen. Für die Umsetzung ähnlicher Projekte sollte dementsprechend eine ausreichende und vermutlich höhere Vergütung der Kooperationskliniken als in unserem Projekt bereits im Vorfeld abgestimmt werden.

### Datenherkunft und -struktur

Aus den Kliniken wurden sehr heterogene Daten ausgeleitet. Relevante Daten aus der Notaufnahmeversorgung lagen unterschiedlich strukturiert und meist in mehreren IT-Systemen vor.

Behandlungsdaten im Notaufnahmekontext sind aufgrund der Vielfalt an Vergütungssystemen an der Schnittstelle zwischen ambulantem und stationärem Sektor grundsätzlich inhomogen. Die Datenerhebung wird unter anderem davon beeinflusst, ob ein Fall ambulant über die KVen, ambulant direkt mit den Krankenkassen oder als vollstationärer Fall abgerechnet wird [12]. Als herausfordernd erwies sich die konkrete Falldefinition auf den unterschiedlichen Ebenen. In der Notaufnahme ist damit ein einzelner Patientenkontakt gemeint, aus Sicht des Controllings kann ein Abrechnungsfall auch mehrere Notaufnahmebesuche enthalten [10]. Dann lassen sich bestimmte Informationen nur noch dem Fall, nicht aber dem konkreten Patientenkontakt in der Notaufnahme zuordnen (z. B. sind Diagnosen im Fall der KV-Abrechnung quartalsbezogen). Eine eindeutige Identifikationsnummer pro Notaufnahmekontakt war nicht überall vorhanden. Auch über die Kombination mehrerer Variablen (z. B. quartalsweise Fallnummer plus Aufnahmedatum) konnte nicht immer eine Eindeutigkeit hergestellt werden.

So war und ist die Bildung eines harmonisierten und plausiblen Datensatzes aus verschiedenen Datenquellen eine große Herausforderung. Ähnliche Herausforderungen wurden selbst bei Krankenkassendaten beschrieben [17], bei denen grundsätzlich eine weitgehende Standardisierung vermutet wird.

### Ausblick

Trotz mehrerer hier beschriebener Einschränkungen hat INDEED neue methodisch-konzeptionelle Standards entwickelt, die dem Anspruch einer Machbarkeitsstudie für ähnliche Versorgungsforschungsprojekte gerecht werden. Notaufnahmedaten liegen in INDEED patientenkontaktbezogen vor, welche so einen Mehrwert gegenüber Abrechnungsdaten darstellen. Dort sind Notaufnahmebehandlungen oft nicht eindeutig identifizierbar. Die Datenverknüpfung mit ambulanten Versorgungsdaten bietet darüber hinaus die Möglichkeit zur Analyse sektorenübergreifender Versorgungsverläufe.

## Fazit für die Praxis

- Die Einführung einer einheitlichen administrativen Dokumentation für alle Notaufnahmebehandlungen sollte angestrebt werden, unabhängig von Kostenträger und Abrechnungsmodus.
- Für medizinische Daten könnte ein *obligatorisch* zu erhebender einheitlicher Kerndatensatz zielführend sein.
- Die Datenausleitung sollte auf wenige, klar definierbare und vergleichbar dokumentierte Daten beschränkt werden, die in möglichst allen Kliniken vorhanden sind. Vorhandene Datensatzbeschreibungen sind zu übernehmen.
- Datenausleitungen in mehreren Tabellen erfordern eine eindeutige Zuordnung der enthaltenen Informationen zu den Fällen.
- Personelle und zeitliche Ressourcen sollten großzügig geplant werden.
- Es sollte gut abgewogen werden, an welcher Stelle der Aufwand für die Optimierung der Datenqualität am sinnvollsten erscheint.
- Ein stetiger interprofessioneller Austausch zwischen den koordinierenden Wissenschaftlern und den Kooperationspartnern ist auf allen Ebenen und von Projektbeginn an unerlässlich.

## References

[1] Australian Institute of Health and Welfare (2015). Emergency department care 2014–15: Australian hospital statistics.

[2] Bernstein SL, Aronsky D, Duseja R (2009). The effect of emergency department crowding on clinically oriented outcomes. Acad Emerg Med.

[3] Bethge N, Fischer-Rosinský A, Zimmermann G (2019). Entwicklung von Datenschutzkonzepten zur Verknüpfung von Routinedaten aus Notaufnahmen mit Routinedaten der Kassenärztlichen Vereinigungen (KVen) im INDEED-Projekt.

[4] Brammen D, Greiner F, Kulla M (2020). Das AKTIN-Notaufnahmeregister – kontinuierlich aktuelle Daten aus der Akutmedizin. Med Klin Intensivmed Notfmed.

[5] Deutsche Krankenhausgesellschaft (2017). Daten nach § 21 KHEntgG: Version 2018 für das Datenjahr 2017.

[6] Deutsche Krankenhausgesellschaft (2018). Datenübermittlung nach § 301 Abs. 3 SGB V.

[7] Eichler M, Schmitt J, Schuler M (2019). Die Dauer von Ethikvoten in Deutschland – am Beispiel einer nicht-interventionellen Beobachtungsstudie mit 44 teilnehmenden Zentren (PROSa). ZEFQ.

[8] Fischer-Rosinsky A, Ebert G, Greiner F (2018). Datenschutzkonformes pseudonymes Data-Linkage von Daten aus Notaufnahmen und der kassenärztlichen Versorgung im Projekt INDEED.

[9] Fischer-Rosinský A, Slagman A, King R (2021). INDEED – Utilization and cross-sectoral patterns of care for patients admitted to emergency departments in Germany: rationale and study design. Front Public Health.

[10] GKV-Spitzenverband (2017). Fallpauschalensystem für Krankenhäuser für das Jahr 2018.

[11] Greiner F, Brammen D, Kulla M (2018). Standardisierte Erhebung von Vorstellungsgründen in der Notaufnahme. Med Klin Intensivmed Notfallmed.

[12] Greiner F, Slagman A, Stallmann C (2020). Routinedaten aus Notaufnahmen: Unterschiedliche Dokumentationsanforderungen, Abrechnungsmodalitaten und Datenhalter bei identischem Ort der Leistungserbringung. Gesundheitswesen.

[13] INDEED-Projekt (2021) Hinweise zum Datenschutz und Umsetzung in INDEED. https://versorgungsforschung.charite.de/forschung/ressourcen/indeed. Zugegriffen: 29. Juli 2021

[14] Kassenärztliche Bundesvereinigung (2017). Richtlinie der Kassenärztlichen Bundesvereinigung nach § 75 Absatz 7 SGB V zur Vergabe der Arzt‑, Betriebsstätten- sowie der Praxisnetznummern.

[15] Kulla M, Baacke M, Schöpke T (2014). Kerndatensatz „Notaufnahme“ der DIVI. Notfall Rettungsmed.

[16] Lucas B, Brammen D, Schirrmeister W (2019). Anforderungen an eine nachhaltige Standardisierung und Digitalisierung in der klinischen Notfall- und Akutmedizin. Unfallchirurg.

[17] March S, Andrich S, Drepper J (2019). Gute Praxis Datenlinkage (GPD). Gesundheitswesen.

[18] Mockel M, Reiter S, Lindner T (2020). „Triagierung“ – Ersteinschätzung von Patienten in der zentralen Notaufnahme. Med Klin Intensivmed Notfmed.

[19] Mockel M, Searle J, Muller R (2013). Chief complaints in medical emergencies: Do they relate to underlying disease and outcome? The Charite Emergency Medicine Study (CHARITEM). Eur J Emerg Med.

[20] Pines JM, Hilton JA, Weber EJ (2011). International perspectives on emergency department crowding. Acad Emerg Med.

[21] Schöpke T, Dodt C, Brachmann M (2014). Statusbericht aus deutschen Notaufnahmen. Ergebnisse der DGINA-Mitgliederbefragung 2013. Notfall Rettungsmed.

[22] Schöpke T, Plappert T (2011). Kennzahlen von Notaufnahmen in Deutschland. Notf Rettungsmed.

[23] Trzeczak S (2013). Überfüllte Notaufnahme. Ursachen, Folgen und Lösungen. Notfall Rettungsmed.

